# Supplementary material for: Polymorphism in the Chloroplast ATP Synthase Beta-Subunit Is Associated with a Maternally Inherited Enhanced Cold Recovery in Cucumber
Source: Plants (Basel). 2021 May 29;10(6):1092. doi: 10.3390/plants10061092 (PMC8226925; doi:10.3390/plants10061092)
Supplement: Supplementary file 1 [file plants-10-01092-s001.zip › plants-1193571-supplementary/Supplemental/Supplemental Materials_ColdCukeManuscript.pdf]

Supplemental Materials

Figure S1. Reciprocal hybrids of DH 'Chipper' (CH) with DH 'Straight 8' (ST8). Plants were grown in a greenhouse at 28 °C for 10 days then plants were either treated at 4 °C for 5.5 hours at a light intensity of 270 $\mu mol \cdot s^{-1} \cdot m^{-2}$ (Cold Treated) or remained in the greenhouse (Control) then were grown for 14 days after treatment (DAT).

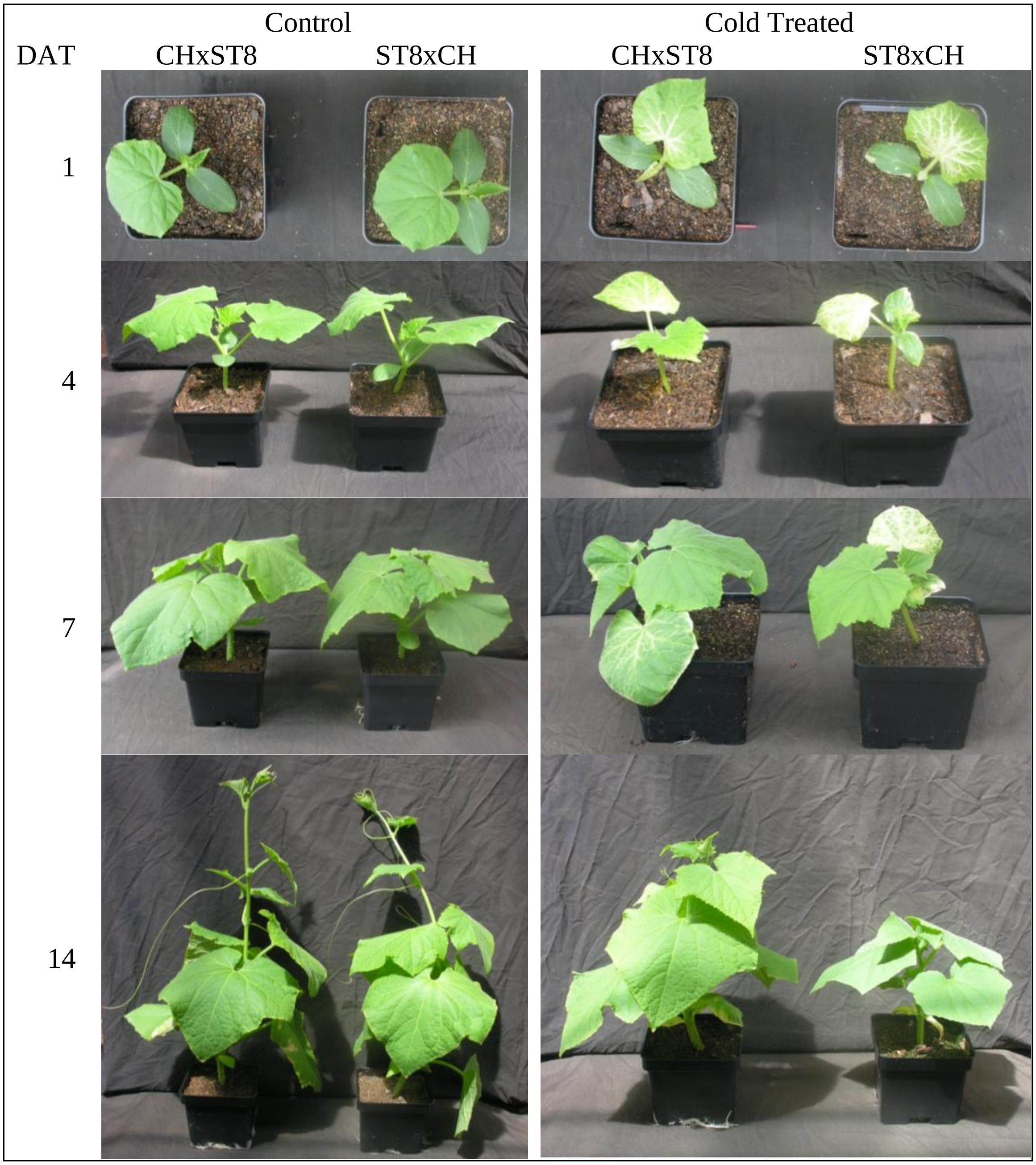

Figure S2. Reciprocal hybrids of DH 'Chipper' (CH) with DH 'Marketmore 76'(MM). Plants were grown in a greenhouse at 28 °C for 10 days then plants were either treated at 4 °C for 5.5 hours at a light intensity of 270 µmol·s^{-1}·m^{-2} (Cold Treated) or remained in the greenhouse (Control) then were grown for 14 days after treatment (DAT).

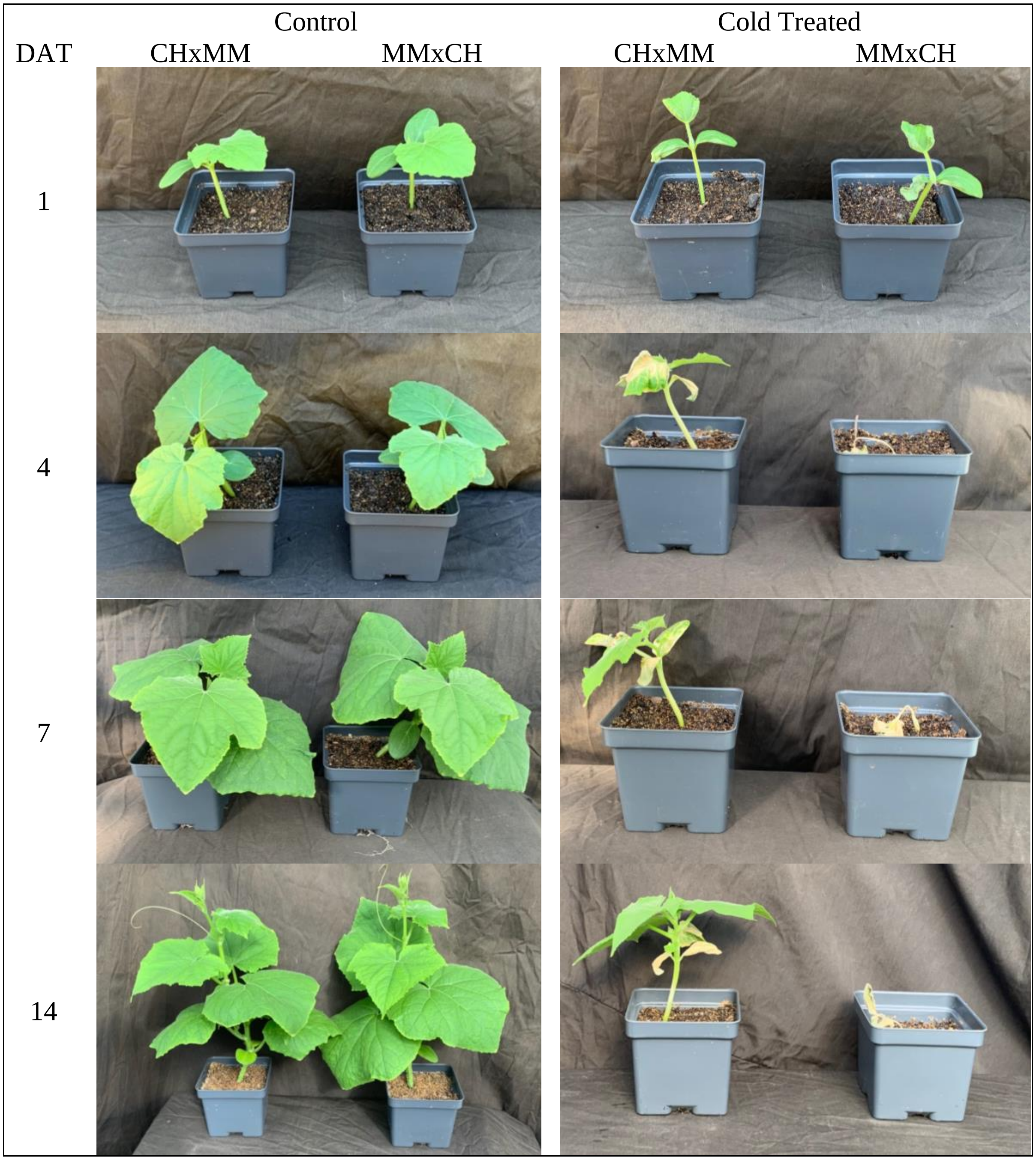

Table S1. Sample-level read mapping statistics for all RNA samples from reciprocal hybrids across the three timepoints, before cold treatment (A), during cold treatment (B), and after cold treatment (C).

Timepoint	Hybrid	Rep	Raw Reads	Aligned (Primary)	%	Unaligned	%
**A**	CHxST8	1	48382008	47402358	94.8	979650	2.0
**A**	CHxST8	2	58281050	57153971	95.4	1127079	1.9
**A**	CHxST8	3	55900324	54771743	95.3	1128581	2.0
**A**	ST8xCH	1	44296336	43375342	95.0	920994	2.0
**A**	ST8xCH	2	47112746	46157371	95.1	955375	2.0
**A**	ST8xCH	3	48627646	47665604	95.1	962042	1.9
**A**	CHxMM	1	43186892	42382311	95.3	804581	1.8
**A**	CHxMM	2	45450896	44469446	95.1	981450	2.1
**A**	CHxMM	3	48484250	47597575	95.4	886675	1.8
**A**	MMxCH	1	55467836	54367700	95.3	1100136	1.9
**A**	MMxCH	2	45943032	45011519	95.2	931513	2.0
**A**	MMxCH	3	50729906	49632461	95.1	1097445	2.1
**B**	CHxST8	1	52786760	51733438	94.8	1053322	1.9
**B**	CHxST8	2	53264846	52229008	95.1	1035838	1.9
**B**	CHxST8	3	50397116	49317813	94.7	1079303	2.1
**B**	ST8xCH	1	50780626	49708035	94.6	1072591	2.0
**B**	ST8xCH	2	47507364	46545182	94.8	962182	2.0
**B**	ST8xCH	3	47364754	46385917	94.7	978837	2.0

**B**	CHxMM	1	47330988	46362135	94.9	968853	2.0
**B**	CHxMM	2	51373384	50377544	95.0	995840	1.9
**B**	CHxMM	3	51411672	50398771	95.1	1012901	1.9
**B**	MMxCH	1	49554954	48492225	94.8	1062729	2.1
**B**	MMxCH	2	43935794	43025715	95.0	910079	2.0
**B**	MMxCH	3	42765302	41860137	95.0	905165	2.1
**C**	CHxST8	1	53057164	51711968	93.8	1345196	2.4
**C**	CHxST8	2	53245150	51872185	93.7	1372965	2.5
**C**	CHxST8	3	5165553	50371192	93.9	1284342	2.4
**C**	ST8xCH	1	48531214	46955212	92.6	1576002	3.1
**C**	ST8xCH	2	48950320	47186828	92.2	1763492	3.4
**C**	ST8xCH	3	48601256	47123416	93.0	1477840	2.9
**C**	CHxMM	1	44154472	43130211	94.1	1024261	2.2
**C**	CHxMM	2	47049986	45997744	94.6	1052242	2.2
**C**	CHxMM	3	52614062	51520384	94.8	1093678	2.0
**C**	MMxCH	1	44998966	43633416	93.2	1395550	3.0
**C**	MMxCH	2	45989974	44686221	93.8	1303753	2.7
**C**	MMxCH	3	46840436	45355438	93.1	1484998	3.0

Table S2. Gene Ontology (GO) terms enriched within the set of differentially expressed genes (DEGs) common to both reciprocal hybrid pairs, MMxCH versus CHxMM and ST8xCH versus CHxST8, 24 hours after onset of cold treatment. Reference gene set (Ref) included all genes detected within the experiment. Significance was tested based on Fisher's exact test and p-value was adjusted based on false discovery rate (FDR) correction.

**Domain***	**GO Term**	**Ref (#/20953)**	**Genes (#/3998)**	**Adjusted p-value**
BP	fatty acid metabolic process (GO:0006631)	145	54	2.30E-02
BP	mitotic cell cycle (GO:0000278)	190	65	3.29E-02
BP	cell cycle (GO:0007049)	331	105	6.30E-03
BP	carboxylic acid metabolic process (GO:0019752)	654	183	7.30E-03
BP	organic acid metabolic process (GO:0006082)	667	186	4.97E-03
BP	oxoacid metabolic process (GO:0043436)	665	184	6.52E-03
BP	small molecule metabolic process (GO:0044281)	1076	271	1.98E-02
BP	metabolic process (GO:0008152)	7310	1565	4.69E-04
BP	organic substance metabolic process (GO:0071704)	6170	1320	5.71E-03
BP	primary metabolic process (GO:0044238)	5806	1234	2.37E-02
BP	cellular metabolic process (GO:0044237)	5814	1235	2.19E-02
BP	biological_process (GO:0008150)	11859	2484	1.89E-07
BP	cellular process (GO:0009987)	8671	1809	5.95E-03
CC	90S preribosome (GO:0030686)	29	16	4.94E-02
CC	microbody (GO:0042579)	88	39	2.74E-03
CC	peroxisome (GO:0005777)	88	39	2.57E-03
CC	preribosome (GO:0030684)	79	35	4.40E-03

CC	microtubule (GO:0005874)	128	52	1.27E-03
CC	supramolecular polymer (GO:0099081)	133	53	1.60E-03
CC	polymeric cytoskeletal fiber (GO:0099513)	133	53	1.47E-03
CC	supramolecular fiber (GO:0099512)	133	53	1.36E-03
CC	microtubule cytoskeleton (GO:0015630)	156	60	1.03E-03
CC	plastid stroma (GO:0009532)	195	67	3.22E-03
CC	nucleolus (GO:0005730)	202	69	3.14E-03
CC	chloroplast stroma (GO:0009570)	191	65	4.86E-03
CC	supramolecular complex (GO:0099080)	189	62	1.45E-02
CC	cytoskeleton (GO:0005856)	227	69	3.67E-02
CC	intracellular non-membrane-bounded organelle (GO:0043232)	1148	310	5.47E-05
CC	non-membrane-bounded organelle (GO:0043228)	1148	310	2.73E-05
CC	cytoplasm (GO:0005737)	5064	1092	1.89E-03
CC	organelle (GO:0043226)	6757	1435	1.05E-03
CC	intracellular organelle (GO:0043229)	6751	1433	1.09E-03
CC	intracellular anatomical structure (GO:0005622)	8106	1712	1.66E-04
CC	membrane-bounded organelle (GO:0043227)	6291	1323	5.79E-03
CC	intracellular membrane-bounded organelle (GO:0043231)	6211	1305	7.08E-03
CC	cellular anatomical entity (GO:0110165)	12076	2477	1.08E-04
CC	cellular component (GO:0005575)	12105	2481	8.41E-05
MF	microtubule motor activity (GO:0003777)	50	25	2.03E-02
MF	microtubule binding (GO:0008017)	114	46	5.71E-03
MF	tubulin binding (GO:0015631)	125	47	1.59E-02

MF	ATP binding (GO:0005524)	1695	423	1.03E-04
MF	adenyl nucleotide binding (GO:0030554)	1750	433	1.18E-04
MF	adenyl ribonucleotide binding (GO:0032559)	1739	430	1.32E-04
MF	purine ribonucleoside triphosphate binding (GO:0035639)	1900	468	8.75E-05
MF	purine ribonucleotide binding (GO:0032555)	1944	475	1.20E-04
MF	purine nucleotide binding (GO:0017076)	1962	479	1.23E-04
MF	small molecule binding (GO:0036094)	2384	581	1.14E-05
MF	nucleotide binding (GO:0000166)	2212	537	5.76E-05
MF	nucleoside phosphate binding (GO:1901265)	2212	537	5.19E-05
MF	carbohydrate derivative binding (GO:0097367)	1994	484	1.39E-04
MF	ribonucleotide binding (GO:0032553)	1971	478	1.59E-04
MF	anion binding (GO:0043168)	2351	569	3.63E-05
MF	DNA binding (GO:0003677)	1446	346	1.32E-02
MF	organic cyclic compound binding (GO:0097159)	4919	1125	3.26E-07
MF	transferase activity (GO:0016740)	2720	622	1.99E-03
MF	heterocyclic compound binding (GO:1901363)	4908	1122	2.88E-07
MF	ion binding (GO:0043167)	4172	934	1.44E-04
MF	catalytic activity (GO:0003824)	6793	1506	1.29E-07
MF	binding (GO:0005488)	7254	1580	1.61E-06

*BP, CC, and MF represent biological process, cellular component, and molecular function domains, respectively.
